# Comparison of Laparoscopic eTEP-RS/TAR and IPOM Techniques for Ventral Hernia Repair

**DOI:** 10.3389/jaws.2025.14176

**Published:** 2025-04-25

**Authors:** Yaninee Anusitviwat, Siripong Cheewatanakornkul, Kamthorn Yolsuriyanwong, Somrit Mahattanobon, Suphawat Laohawiriyakamol, Piyanun Wangkulangkul

**Affiliations:** Department of Surgery, Faculty of Medicine, Prince of Songkla University, Hat Yai, Songkhla, Thailand

**Keywords:** eTEP, enhanced-view totally extraperitoneal, ventral hernia repair, retromuscular repair, IPOM, recurrence

## Abstract

**Background:**

The laparoscopic intraperitoneal onlay mesh (IPOM) technique has been widely used for ventral hernia repair; however, concerns regarding mesh-related complications have led to the development of alternative approaches. The enhanced-view totally extraperitoneal (eTEP) technique has emerged as a promising alternative, offering improved anatomical restoration and reduced postoperative morbidity. This study compares the clinical outcomes of eTEP and IPOM for ventral hernia repair.

**Methods:**

A retrospective cohort study was conducted at a tertiary referral centre in Thailand. Patients who underwent laparoscopic ventral hernia repair using either eTEP or IPOM between January 2016 and December 2021 were included. Demographic data, hernia characteristics, perioperative variables, and postoperative outcomes were analysed. Statistical comparisons were performed using parametric and non-parametric tests, with a significance threshold of *p* < 0.05.

**Results:**

A total of 70 patients were included, with 32 undergoing eTEP and 38 undergoing IPOM. Both groups were comparable in baseline characteristics, with most cases classified as incisional hernias. The mean operative time was significantly longer in the eTEP group (360 vs. 240 min, *p* < 0.001). Subgroup analysis showed significantly lower postoperative pain scores at 12 and 24 h in the eTEP-RS and eTEP-TAR groups compared to the IPOM group (*p* < 0.001). The mean VAS scores at 12 h were 4 (eTEP-RS), 3 (eTEP-TAR), and 7.5 (IPOM), while at 24 h, they decreased to 2 (eTEP-RS), 2 (eTEP-TAR), and 4 (IPOM). Complication rates were comparable between groups; however, minor bowel injury was reported in some IPOM cases. The one-year recurrence rate was 3.1% for eTEP and 7.9% for IPOM (p = 0.620), increasing to 6.2% and 15.8% at 2 years, respectively (p = 0.275).

**Conclusion:**

Laparoscopic eTEP is a safe and effective alternative to IPOM for medium to large ventral hernias, demonstrating lower postoperative pain and recurrence rates. However, its technical complexity and longer operative time highlight the importance of careful patient selection and surgical expertise. Further prospective studies with larger sample sizes are needed to validate these findings and optimise clinical outcomes.

## Introduction

The laparoscopic intraperitoneal onlay mesh (IPOM) technique has become the standard approach for ventral hernia repair following the seminal work of LeBlanc et al. in 1993. This method has been shown to facilitate enhanced recovery and a reduction in wound-related complications [1, 2] However, it also has some drawbacks. The placement of intraperitoneal mesh can lead to adhesions, fistula formation, and migration, which may cause serious complications. Additionally, mesh fixation methods, while necessary for stability, have been linked to higher levels of postoperative pain and an increased need for reoperations, raising concerns about its long-term effectiveness and patient outcomes [2, 3].

Ventral hernia repair has undergone significant advancements over the past two decades, with minimally invasive techniques increasingly favouring the intraperitoneal onlay mesh (IPOM) approach. More recently, there has been a paradigm shift towards a patient-centred model, prioritising quality of life and mitigating surgery-related morbidity. This evolution encompasses strategies aimed at restoring abdominal wall function, reducing recurrence rates, and minimising postoperative complications, including seroma formation, bulging, and chronic pain [4–7]. To minimise the risks associated with intraperitoneal mesh placement, several minimally invasive techniques have been developed, including the enhanced-view totally extraperitoneal (eTEP) approach, Mini- or Less-Open Sublay Operation (MILOS), and laparoscopic subcutaneous onlay mesh (SCOM) [8–11]. Among these, the eTEP technique has emerged as a widely favoured alternative, providing enhanced anatomical visualisation and a lower incidence of postoperative complications, thereby improving surgical outcomes and patient recovery.

The enhanced-view totally extraperitoneal (eTEP) technique was first introduced in 2012 for laparoscopic inguinal hernia repair and later refined for ventral hernia reconstruction. This approach was developed to mitigate the risks associated with intraperitoneal mesh placement and to optimise surgical outcomes. By positioning the mesh within the retro-rectus space, eTEP enables abdominal wall reinforcement while preserving the integrity of the peritoneal cavity, thereby reducing the likelihood of mesh-related complications [12] The integration of the enhanced-view totally extraperitoneal (eTEP) approach with transversus abdominis muscle release has yielded highly favourable outcomes. This technique promotes the restoration of abdominal wall anatomy and function while minimising the risk of mesh-related complications by preventing direct contact with intra-abdominal organs [11, 13].

The enhanced-view totally extraperitoneal (eTEP) approach is increasingly utilised for abdominal wall hernia repair. However, early evidence remains limited, with most studies focusing on short-term outcomes and the learning curve [11, 14] Current literature predominantly originates from high-volume centres, highlighting the need for multicentre studies to establish long-term efficacy [11, 15]. Despite the shift towards minimally invasive techniques, eTEP remains largely limited among specialised centres. Comparative studies evaluating eTEP against IPOM and IPOM Plus are primarily based on small sample sizes and focus on patients with smaller hernias. Most analyses compare early postoperative recovery and short-term outcomes [16–18].

The adoption of laparoscopic enhanced-view totally extraperitoneal (eTEP) repair is increasing across Southeast Asia; however, the available literature predominantly comprises reports on initial experiences [19]. In Thailand, laparoscopic hernia repair is primarily conducted by minimally invasive surgeons or within tertiary healthcare institutions. As this paradigm shift progresses, further high-quality research is required to develop evidence-based guidelines for the optimal selection between laparoscopic eTEP and intraperitoneal onlay mesh (IPOM) in clinical practice.

This study assesses our initial experience and postoperative outcomes of laparoscopic ventral hernia repair, comparing the enhanced-view totally extraperitoneal (eTEP-RS/TAR) approach with the intraperitoneal onlay mesh (IPOM) technique. All procedures were performed over a five-year period at a single tertiary centre.

## Materials and Methods

A retrospective cohort study was conducted at Prince of Songkla University, a tertiary referral centre for hernia management in southern Thailand. The study included patients diagnosed with ventral hernia (both primary and incisional) [20] who underwent laparoscopic retromuscular repair using either the enhanced-view totally extraperitoneal (eTEP) approach or the intraperitoneal onlay mesh (IPOM) technique between January 2016 and December 2021. Ethical approval was obtained from the hospital’s ethics committee prior to patient enrolment. A total of 70 patients were included and stratified into two groups based on the surgical approach: laparoscopic eTEP and IPOM.

### Surgical Technique

#### eTEP Approach

The enhanced-view totally extraperitoneal (eTEP) procedure was conducted using a standardised dual-surgeon setup. The patient was positioned in a supine posture, with the arms aligned alongside the body. Depending on the hernia location, slight extension of the back or hips was applied to optimise surgical exposure. Access to the retrorectus space was facilitated using a balloon spacemaker, with port placement determined based on the hernia defect size, location, and any previous surgical scars. Midline crossover was performed through an intact anatomical region, and transversus abdominis release (TAR) was undertaken in cases where adequate mesh overlap was unachievable or when defect closure resulted in excessive tension. Preservation of the linea alba and neurovascular bundles was prioritised throughout the procedure. The hernia defect was meticulously repaired, followed by reconstruction of the linea alba using 0 Stratafix^TM^ absorbable sutures, with additional suture reinforcement applied along pre-existing scar lines. The posterior fascial defect was subsequently closed prior to mesh placement, ensuring a minimum overlap of 5 cm in all directions. Histoacryl® glue was utilised for mesh fixation, and surgical drains were routinely placed in accordance with postoperative management protocols.

#### IPOM Approach

The conventional intraperitoneal onlay mesh (IPOM) technique was performed by surgeons with a minimum of 5 years’ experience in laparoscopic surgery. The patient was positioned in a supine position, with the arms placed bilaterally along the body. The key procedural steps included establishing pneumoperitoneum, performing adhesiolysis, and placing a composite mesh with a minimum overlap of 5 cm beyond the hernia defect, without primary defect closure. The mesh was secured using sutures at 2–4 fixation points, with tackers providing additional reinforcement to ensure stability.

### Variables

- Patient Demographics: Age, gender, body mass index (BMI, kg/m^2^), American Society of Anesthesiologists (ASA) classification, comorbidities and hernia risk factors, which assesses the patient’s overall health status and perioperative risk.
- Hernia Symptoms: The presence of pain, urinary disturbances, obstructive symptoms, and general discomfort.
- Radiological Data: Imaging findings from computed tomography (CT) scans, including hernia classification as per the European Hernia Society (EHS), rectus hernia defect size (cm^2^), and abdominis muscle dimensions.
- Perioperative Variables: The assessed parameters included operative time (minutes), total blood loss, placement of drains, mesh type, and mesh area (cm^2^).
- Postoperative Variables: Postoperative pain was evaluated using the Visual Analogue Scale (VAS), ranging from 0 to 10, with pain scores recorded at 4 h intervals up to 12 h postoperatively and every 6 h up to 24 h. Multimodal analgesia, comprising oral analgesics, non-steroidal anti-inflammatory drugs (NSAIDs), and opioids, was administered in all cases unless contraindicated. Postoperative complications included wound morbidities, such as surgical site infection, wound dehiscence, haematoma, and symptomatic seroma. Length of hospital stay and hernia recurrence were systematically monitored over a one-year follow-up period.

### Statistical Analysis

All statistical analyses were performed using R software (R Core Team, 2024). Categorical variables were summarised as frequencies and percentages and compared between groups using either the Chi-square test or Fisher’s exact test, as appropriate. Continuous variables were presented as mean ± standard deviation or median with interquartile range (IQR), depending on the data distribution. Independent t-tests or Mann-Whitney U tests were employed for two-group comparisons, whereas one-way ANOVA was applied for analyses involving three groups. A p-value of < 0.05 was considered indicative of statistical significance.

## Results

### Patient and Hernia Characteristics

Between January 2016 and December 2021, a total of 70 patients were enrolled in the study, with 32 undergoing the eTEP procedure and 38 undergoing the IPOM procedure. Within the eTEP group, 15 patients underwent eTEP-RS, while 17 underwent eTEP-TAR.

Patient demographics, as summarised in Table 1, demonstrated no significant differences between the groups in terms of gender, age, BMI, or ASA classification. The mean (SD) age of the cohort was 62.3 (12.5) years, with a median BMI of 27 kg/m^2^ (IQR: 24–32). The majority of patients (67.1%) were classified as ASA class II. Comorbidities, similarly revealed no statistically significant differences between the groups for most variables. Hypertension (46.9% in the eTEP group versus 63.2% in the IPOM group) and dyslipidaemia (46.9% versus 28.9%) were the most frequently reported comorbid conditions. Diabetes mellitus, acknowledged both as a comorbidity and a recognised risk factor for hernia recurrence, was observed in 25.0% of patients in the eTEP group and 23.7% in the IPOM group. Cardiovascular disease was present in 6.2% of patients undergoing eTEP and 2.6% of those in the IPOM group. Notably, liver disease was significantly more prevalent in the eTEP group (18.8%) compared to the absence of cases in the IPOM group (p = 0.007). Other variables, including previous wound infection, smoking status, steroid use, renal insufficiency, and chronic lung disease, did not demonstrate statistically significant differences between the two groups. Incisional hernias were observed in 55 patients (78.6%), while 14 patients (20%) presented with recurrent ventral hernias. With regard to hernia-related symptoms, pain was the most frequently reported complaint, affecting 40.6% of patients in the eTEP group and 42.1% in the IPOM group.

**TABLE 1:** Patient demographics and hernia classification.

Variables	eTEP (n = 32)	IPOM (n = 38)	*p* value
Patient Demographics: Gender, n (%) Male (n = 15, 21.4%) Female (n = 55, 78.6%)	8 (25.0) 24 (75.0)	7 (18.4) 31 (81.6)	0.707
Age (years), Mean ± SD	62.0 ± 12.7	62.7 ± 12.5	0.820
BMI (kg/m^2^), Median (IQR)	29.5 (23.8, 31.0)	27.0 (24.0, 33.8)	0.832
ASA score, n (%) I II III	1 (3.1) 21 (65.6) 10 (31.2)	1 (2.6) 26 (68.4) 11 (28.9)	1.000
Comorbidities and Risk factors: Hypertension (%) Dyslipidemia (%) Diabetes (%) Previous chemotherapy (%) Smoking/passive smoker (%) Steroid therapy (%) Cardiovascular disease (%) Renal insufficiency (%) Immunosuppression (%) Chronic lung disease (%) Liver disease (%) Previous wound infection (%)	15 (46.9) 15 (46.9) 8 (25.0) 5 (15.6) 2 (6.2) 1 (3.1) 2 (6.2) 9 (28.1) 1 (3.1) 2 (6.2) 6 (18.8) 5 (15.6)	24 (63.2) 11 (28.9) 9 (23.7) 3 (7.9) 0 (0) 0 (0) 1 (2.6) 4 (10.5) 0 (0) 1 (2.6) 0 (0) 2 (5.3)	0.261 0.194 1.000 0.455 0.205 0.457 0.457 0.115 0.457 0.589 0.007 0.234
Hernia Characteristics: Symptoms, n (%) Asymptomatic Symptomatic	19 (59.4) 13 (40.6)	22 (57.9) 16 (42.1)	1.000
Type, n (%) Primary Incisional Recurrent	1 (3.1) 24 (75.0) 7 (21.9)	0 (0) 31 (81.6) 7 (18.4)	0.649
Location (EHS classification), n (%) M1 M2 M3 M4 M5 L1 L2 L3 L4	0 (0) 1 (3.1) 15 (46.9) 7 (21.9) 1 (3.1) 0 (0) 8 (25.0) 0 (0) 0 (0)	0 (0) 3 (7.9) 18 (47.4) 9 (23.7) 1 (2.6) 0 (0) 7 (18.4) 0 (0) 0 (0)	0.922
Defect size (EHS classification), n (%) W1 W2 W3	3 (9.4) 25 (78.1) 4 (12.5)	5 (13.2) 30 (78.9) 3 (7.9)	0.763
Hernia Defect Measurements:, Median (IQR)			
Length (cm) Width (cm)	6.5 (5.0, 8.2) 5.0 (5.0, 7.0)	6.0 (5.0, 8.0) 5.0 (4.0, 7.0)	0.458 0.676
Area (cm^2^)	34.0 (20.0, 56.0)	35.0 (17.0, 53.5)	0.516
Rectus abdominis size (cm), Mean ± SD Right Left	4.0 ± 1.8 4.8 ± 1.3	4.8 ± 1.6 4.4 ± 2.0	0.058 0.338

All hernias were classified according to the European Hernia Society (EHS) guidelines [21]. There were no statistically significant differences between the groups in terms of hernia location, size, or mean defect area. The most frequently observed hernia location in both groups was M3 (umbilical), followed by M4 (infraumbilical) and L2 (flank). According to the EHS hernia defect classification, the majority of hernias were categorised as W2 (4–10 cm) in both groups (78.1% in eTEP vs. 78.9% in IPOM, p = 0.763). The median hernia defect size was comparable, with a length of 6.5 cm (IQR: 5.0–8.2) in the eTEP group versus 6.0 cm (IQR: 5.0–8.0) in the IPOM group (p = 0.458) and a width of 5.0 cm (IQR: 5.0–7.0) vs. 5.0 cm (IQR: 4.0–7.0) (p = 0.676). Similarly, the median hernia area was 34.0 cm^2^ (IQR: 20.0–56.0) in eTEP and 35.0 cm^2^ (IQR: 17.0–53.5) in IPOM, with no significant difference (p = 0.516). The mean rectus abdominis muscle size measured 4.0 ± 1.8 cm (right) and 4.8 ± 1.3 cm (left) in the eTEP group, compared to 4.8 ± 1.6 cm (right) and 4.4 ± 2.0 cm (left) in the IPOM group, with no statistically significant differences (p = 0.058 and p = 0.338, respectively).

### Perioperative Outcomes

Perioperative outcomes are summarised in Table 2. The mean operative time was significantly shorter in the IPOM group (240.0 min) compared to the eTEP group (360.0 min, p < 0.001). However, there were no statistically significant differences between the groups in terms of blood loss. A total of six types of mesh were utilised. In the eTEP group, both composite and uncoated meshes were employed, with a lower mean mesh area of 225.6 cm^2^. Conversely, the IPOM group predominantly utilised composite mesh, which had a larger mean area of 300.0 cm^2^.

**TABLE 2:** Perioperative data and mesh characteristics.

Variables	eTEP (n = 32)	IPOM (n = 38)	*p* value
Perioperative data:			
Operative time (min), Median (IQR)	360.0 (247.5, 497.5)	240.0 (180.0, 300.0)	<0.001
Blood loss (mL), Median (IQR)	10.0 (5.0, 16.2)	10.0 (10.0, 18.8)	0.222
Placement of drain, n (%)	30 (93.8)	3 (7.9)	<0.001
Mesh Characteristics:			
Type of mesh used, n (%) Parietex^TM^ Physiomesh^TM^ Other composite mesh Versatex^TM^ Prolene mesh^TM^ Other Polypropylene soft mesh	2 (6.2) 0 (0) 2 (6.2) 12 (37.5) 8 (25) 8 (25.0)	28 (73.7) 6 (15.8) 3 (7.9) 0 (0) 0 (0) 1 (2.6)	<0.001
Mesh size, Median (IQR) Length (cm) Width (cm)	16.5 (15.0, 24.2) 14.5 (11.5, 15.0)	20.0 (15.0, 20.0) 15.0 (10.0, 15.0)	0.631 0.731
Mesh area (cm^2^), Median (IQR)	225.0 (165.8, 341.2)	300.0 (150.0, 300.0)	0.976

Subgroup analysis, as outlined in Table 3, indicated that the operative time for eTEP-RS remained longer than that of IPOM (320 min vs. 240 min). Blood loss during TAR procedures was minimal, ranging between 5 and 10 mL. In accordance with our institutional protocol, routine drain placement was performed in the eTEP group, particularly in eTEP-TAR cases.

**TABLE 3:** Subgroup Analysis of Hernia Repair Techniques: Perioperative data and Mesh characteristic.

Variables	eTEP-RS (n = 15)	eTEP-TAR (n = 17)	IPOM (n = 38)	*p* value
Perioperative data:				
Operative time (min), Median (IQR)	320 (275, 420)	360 (240, 600)	240 (180, 300)	0.002
Blood loss (mL), Median (IQR)	10.0 (7.5, 20.0)	5.0 (5.0, 10.0)	10.0 (10.0, 18.8)	0.107
Placement of drain, n (%)	13 (86.7)	17 (100)	3 (7.9)	<0.001
Mesh Characteristics:				
Mesh type, n (%) Parietex^TM^ Physiomesh^TM^ Other composite mesh Versatex^TM^ Prolene mesh^TM^ Other Polypropylene soft mesh	0 (0) 0 (0) 2 (13.3) 4 (26.7) 5 (33.3) 4 (26.7)	2 (11.8) 0 (0) 0 (0) 8 (47.1) 3 (17.6) 4 (23.6)	28 (73.7) 6 (15.8) 3 (7.9) 0 (0) 0 (0) 1 (2.6)	<0.001
Mesh size, Median (IQR) Length (cm) Width (cm)	15 (12.5, 16.5) 12 (10, 15)	20 (15, 28) 15 (13, 15)	20 (15, 20) 15 (10, 15)	0.007 0.415
Mesh area (cm^2^), Median (IQR)	169 (150, 240)	280 (225, 450)	300 (150, 300)	0.070

### Postoperative Outcomes

The postoperative variables are summarised in Table 4. In terms of postoperative pain, the IPOM group recorded significantly higher VAS scores at both 12 and 24 h postoperatively compared to the eTEP group (7.5 vs. 4.0 at 12 h and 4.0 vs. 2.0 at 24 h, p < 0.001). However, there were no statistically significant differences between the groups regarding hospital stay duration or overall complication rates. Subgroup analysis, as detailed in Table 5, further demonstrated that the IPOM group exhibited significantly higher mean pain scores at 12 and 24 h compared to the eTEP-TAR group, with values of 7.5 vs. 3.0 at 12 h and 4.0 vs. 2.0 at 24 h, p < 0.001 respectively. Regarding postoperative complications, the overall incidence was low. In the eTEP-RS group, 2 cases (13.3%), and in the eTEP-TAR group, 1 case (5.9%), were reported as symptomatic seromas requiring drainage. No significant surgical site occurrences requiring specific treatment were recorded in either group. In the IPOM group, two cases of serosal tears occurred without full-thickness bowel injury, while three cases of pseudo-recurrence were documented.

**TABLE 4:** Comparison of Postoperative Outcomes Between eTEP and IPOM.

Variables	eTEP (n = 32)	IPOM (n = 38)	*p* value
Post operative pain (VAS score), Median (IQR) at 12 h at 24 h.	4.0 (3.0, 5.2) 2.0 (1.0, 2.0)	7.5 (5.0, 8.0) 4.0 (2.2, 5.0)	<0.001 <0.001
Hospital stays (days), Median (IQR)	4 (3, 7)	4 (2, 5)	0.209
Complications, n (%)	3 (9.4)	5 (13.2)	0.719
Recurrent Rate, n (%) At 1 year At 2 years	1 (3.1) 2 (6.2)	3 (7.9) 6 (15.8)	0.620 0.275
Follow up time (months) Median (IQR) Min-Max	17.1 (5.8, 39.4) 1.08–52.63	10.9 (3.6, 28.3) 0.03–88.44	0.294

**TABLE 5:** Subgroup Analysis of eTEP-RS, eTEP-TAR, and IPOM Outcomes.

Variables	eTEP-RS (n = 15)	eTEP-TAR (n = 17)	IPOM (n = 38)	*p* value
Post operative pain (VAS score), Median (IQR) At 12 h At 24 h	4.0 (4.0, 5.0) 2.0 (1.5, 2.0)	3 (3.0, 5.0) 2 (1.0, 2.0)	7.5 (5.0, 8.0) 4.0 (2.2, 5.0)	<0.001 <0.001
Hospital stays (days), Median (IQR)	4 (3.5, 6.5)	4.0 (3.0, 7.0)	4.0 (2.0, 5.0)	0.423
Complications, n (%)	2 (13.3)	1 (5.9)	5 (13.2)	0.780
Recurrent Rate, n (%) At 1 year At 2 years	0 (0) 1 (6.7)	1 (5.9) 1 (5.9)	3 (7.9) 6 (15.8)	0.804 0.605
Follow up time (months) Median (IQR) Min-Max	18.8 (5.7, 38.9) 1.31–48.23	11.7 (5.9, 38.6) 1.08–52.63	10.9 (3.6, 28.3) 0.03–88.44	0.539

Over the one-year follow-up period, no significant differences in hernia recurrence rates were observed between the groups. In the eTEP-TAR subgroup, one recurrence was reported in a complex mirror L incision, where laparoscopic suturing was insufficient for defect closure. The majority of recurrence cases in both the eTEP and IPOM groups were identified at the two-year follow-up, though statistical comparison was constrained by the small sample size.

## Discussion

This study presents a comparative analysis of the eTEP and IPOM techniques for ventral hernia repair, emphasising distinctions in perioperative and postoperative outcomes. The findings indicate that eTEP was associated with significantly lower postoperative pain scores at both 12 and 24 h compared to IPOM, likely attributable to differences in mesh fixation techniques. Furthermore, eTEP exhibited a lower recurrence rate at both one and two years, reinforcing its efficacy in ventral hernia repair. However, the mean operative time was markedly longer in the eTEP group, reflecting the technical complexity and learning curve associated with the procedure. While complication rates were comparable between the groups, minor bowel injuries were observed in some IPOM cases. These findings suggest that eTEP may offer distinct advantages over IPOM, particularly in reducing postoperative pain and recurrence rates, thereby positioning it as a promising alternative for medium to large ventral hernias.

In Thailand, the increasing prevalence of incisional hernias following previous open surgeries has led to a substantial rise in follow-up cases. Consequently, our study underscores incisional hernia as a significant clinical challenge in southern Thailand. Given the documented advantages of eTEP-TAR, this technique offers a promising approach for optimising surgical outcomes and improving patient care [16, 22, 23]. Laparoscopic eTEP has emerged as a superior alternative to IPOM, particularly for large hernia defects and recurrent cases following previous IPOM repair. Our findings indicate that IPOM was associated with a longer operative time compared to other studies, potentially due to the larger hernia size and the high prevalence of incisional hernias, with recurrence rates reported at 18.4% [24, 25] eTEP-TAR has increasingly superseded IPOM in the management of medium to large hernias, as it enables the placement of a larger mesh area, thereby enhancing surgical outcomes and optimising long-term patient recovery [7, 17, 18, 23, 26] The mean operative time for eTEP-RS and eTEP-TAR was longer than that reported in previous studies, likely reflecting the early learning phase associated with the technique. The eTEP approach requires a highly specialised skill set, encompassing retrorectus space creation, midline crossover, transversus abdominis release (TAR), and laparoscopic suturing, all of which contribute to a steeper learning curve. Consequently, the mean operative times for eTEP-RS and eTEP-TAR were significantly longer than those for IPOM, a trend that is consistent with findings from earlier research [7, 16–18, 22, 27].

Several studies have evaluated postoperative pain following eTEP and IPOM procedures. Existing evidence indicates that mean VAS scores at 12 and 24 h postoperatively were highest in the IPOM group. [4, 7, 18, 22, 27] In our study, although variations in fixation devices were noted, all patients underwent combined fixation, which may have been a contributing factor to increased postoperative pain [4]. Moreover, IPOM was associated with the most prolonged persistence of postoperative pain, a trend that is consistent with our findings [3, 4, 16, 18, 22, 27] Existing literature indicates that mesh fixation techniques may contribute to lower postoperative pain levels in the eTEP-RS and eTEP-TAR groups. This is primarily due to the avoidance of traumatic or aggressive mesh fixation and the strategic positioning of the mesh within the retromuscular layer, which reduces the likelihood of adhesion formation and fistula development [17, 23, 28].

The mean length of hospital stay in the eTEP group was longer than that reported in previous studies [4, 7, 18, 22, 27] This prolonged admission may be attributed to the relative novelty of the eTEP technique at the time of the study. Furthermore, as a significant proportion of patients resided in rural areas, hospitalisation was extended until drain removal and until patients were sufficiently prepared for independent self-care management prior to discharge.

Two cases of serosal injury were identified in the IPOM group, primarily as a consequence of extensive adhesiolysis. However, no major bowel injuries, such as full-thickness perforations, were observed. Postoperative seroma formation has been reported in up to 30% of cases [23, 29]. In our study, seroma was diagnosed in 25% of eTEP-RS cases and 12% of eTEP-TAR cases, which may be attributed to the creation of an extensive retromuscular space during the procedure. Furthermore, the incidence of seroma could be influenced by the choice of mesh, particularly polyester-based variants such as Parietex™ and Versatex™. Mild seroma-related symptoms generally resolved over time without the need for surgical intervention. Based on our observations, seroma regression occurred within 3–5 days in eTEP-TAR cases, and routine drain placement did not appear to prevent postoperative seroma formation. Given the low risk of bleeding associated with the eTEP technique, routine drain placement may not be necessary. Moreover, variations were noted in the administration of prolonged oral prophylactic antibiotics in selected cases; however, no significant postoperative infections or requirements for additional interventions were documented. It is important to acknowledge that the data concerning this practice are limited, due to the inherent constraints and potential inconsistencies associated with retrospective data collection.

Although eTEP presents a steep learning curve, its complication rates were lower than those reported for IPOM, consistent with findings from previous studies [30]. This may be attributed to surgeons’ prior experience with open TAR procedures and their proficiency in eTEP for groin hernia repair. Additionally, cadaveric training and a structured dual-surgeon approach have proven invaluable in facilitating the safe adoption of laparoscopic eTEP-TAR, particularly during the early learning phase.

The recurrence rate of IPOM in our study was 7.9% at 1 year and 15.8% at 2 years. Reported recurrence rates for IPOM vary between 3.5% and 24%, depending on factors such as defect closure, mesh type, and fixation technique [31–33]. Several factors may have contributed to the recurrence rate observed in our study, including defect size, the extent of mesh overlap, and the specific type of mesh used. In cases of medium to large defect sizes, IPOM demonstrated a slightly higher recurrence rate compared to eTEP-RS and eTEP-TAR. A review of operative records indicated that recurrences were most frequently observed in W2 (medium) and W3 (large) defect sizes, suggesting that a 5 cm mesh overlap may be insufficient for preventing recurrence, particularly in larger defects, as outlined in Supplementary Table S1 [34]. Larger defects necessitate increased mesh overlap; however, they also pose challenges, such as shrinkage and folding due to restricted intraperitoneal space, which may compromise mesh integration and long-term durability. At the two-year follow-up, the increased recurrence rate in the IPOM group may be attributed to factors such as tissue remodelling and suboptimal mesh integration. Notably, Parietex™, a non-absorbable polyester mesh with a collagen coating, features a medium to large pore size that facilitates tissue ingrowth; however, experimental animal model studies have associated Parietex™ with an increased risk of shrinkage [35]. Additionally, the recall of Physiomesh™ in 2016, which occurred during the study period, may have contributed to the elevated recurrence rates observed [36]. Furthermore, the fixation method plays a crucial role in long-term surgical outcomes. Evidence suggests that fascial closure can significantly reduce recurrence rates and postoperative bulging, emphasising its importance in enhancing the durability of hernia repair compared to conventional IPOM [28, 37]. Nevertheless, due to the limited sample size, establishing a definitive causal relationship remains challenging.

In low- to middle-income countries, open hernia repair remains widely utilised, often leading to larger defect sizes and, in some cases, hernia formation in atypical anatomical sites. The laparoscopic eTEP-RS and eTEP-TAR approaches provide significant advantages in managing these complex cases, demonstrating superior effectiveness compared to IPOM, as they are associated with reduced postoperative pain, fewer complications, and comparable or potentially improved recurrence rates. [18, 22] A notable advantage of eTEP-RS and eTEP-TAR over IPOM is the ability to employ non-barrier mesh, which serves as a more cost-effective alternative to the composite mesh required for intraperitoneal placement. While eTEP procedures are associated with longer operative durations, a steeper learning curve, and increased technical complexity, their clinical benefits make them a valuable approach for ventral hernia repair [38].

## Strengths and Limitation

This study offers a comparative analysis of the enhanced-view totally extraperitoneal (eTEP) and intraperitoneal onlay mesh (IPOM) techniques, addressing the limited literature on their clinical outcomes. A five-year data collection period strengthens the evaluation of long-term patient outcomes and recurrence rates. Conducted in a real-world tertiary care setting, the findings hold broad clinical relevance. However, limitations include a small sample size of laparoscopic eTEP cases due to the introduction of robotic eTEP in December 2021 and the retrospective nature of the study, which may limit the ability to fully assess the impact of specific risk factors on outcomes. To further validate these findings and refine clinical decision-making, future research should incorporate larger cohorts, propensity-matched analysis, and extended follow-up periods.

## Conclusion

This study indicates that laparoscopic eTEP may serve as a safe and effective alternative to conventional laparoscopic IPOM for medium- to large-sized hernias, offering advantages such as reduced postoperative pain and a lower incidence of mesh-related complications. However, the eTEP technique presents notable technical challenges and necessitates a steep learning curve, requiring a thorough understanding of anatomy alongside advanced surgical expertise. Furthermore, eTEP is associated with a prolonged operative duration compared to IPOM, underscoring the need for meticulous patient selection to optimise outcomes. For small-sized hernias, IPOM plus is a preferable option for experienced laparoscopic surgeons compared to standard IPOM, given its relatively straightforward approach and acceptable recurrence rates. Key modifiable elements, such as defect closure, a comprehensive understanding of mesh properties, and continuous advancements in surgical techniques, play a crucial role in optimising surgical outcomes.

## Data Availability

The raw data supporting the conclusions of this article will be made available by the authors, without undue reservation.
