# Supplementary material for: Comparison of Laparoscopic eTEP-RS/TAR and IPOM Techniques for Ventral Hernia Repair
Source: J Abdom Wall Surg. 2025 Apr 25;4:14176. doi: 10.3389/jaws.2025.14176 (PMC12061722; doi:10.3389/jaws.2025.14176)

**Supplement table S1 Recurrent rate by defect size**

| Defect size | Adequate mesh overlap (5cm) | Inadequate mesh overlap |
| --- | --- | --- |
| W1 | 0/0 (0%) | 1/7 (14.3%) |
| W2 | 2/19 (10.5%) | 5/36 (13.9%) |
| W3 | 1/1 (100%) | 1/6 (16.7%) |
| Total | 3 (14.3) | 7 (14.3) |

**Calculation Logic:**

1. The table presents recurrence rates for different defect sizes (W1, W2, W3) based on mesh overlap adequacy:
   - Adequate mesh overlap: Defined as at least 5 cm beyond the hernia defect.
   - Inadequate mesh overlap: Defined as less than 5 cm beyond the hernia defect.
2. Recurrence Rate Calculation:
   - The recurrence rate is determined using the formula:


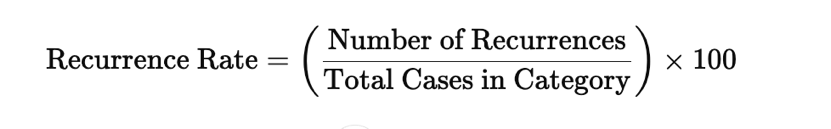

Supplement: Supplementary file 1 [file Table1.docx]
